# *Planococcus maritimus* ML1206 Isolated from Wild Oysters Enhances the Survival of *Caenorhabditis elegans* against *Vibrio anguillarum*

**DOI:** 10.3390/md19030150

**Published:** 2021-03-12

**Authors:** Ying-Xiu Li, Nan-Nan Wang, Yan-Xia Zhou, Chun-Guo Lin, Jing-Shan Wu, Xin-Qi Chen, Guan-Jun Chen, Zong-Jun Du

**Affiliations:** 1 Marine College, Shandong University, Weihai 264209, China; yingxiuli@sdu.edu.cn (Y.-X.L.); wangnan2016@mail.sdu.edu.cn (N.-N.W.); zhouyx@sdu.edu.cn (Y.-X.Z.); linchunguo@mail.sdu.edu.cn (C.-G.L.); wujingshan@mail.sdu.edu.cn (J.-S.W.); chenxinqi@mail.sdu.edu.cn (X.-Q.C.); 2 State Key Laboratory of Microbial Technology, Shandong University, Jinan 250100, China

**Keywords:** marine probiotics, *Planococcus maritimus*, *Vibrio anguillarum*, *Caenorhabditis elegans*, real-time PCR

## Abstract

With the widespread occurrence of aquaculture diseases and the broad application of antibiotics, drug-resistant pathogens have increasingly affected aquatic animals’ health. Marine probiotics, which live under high pressure in a saltwater environment, show high potential as a substitute for antibiotics in the field of aquatic disease control. In this study, twenty strains of non-hemolytic bacteria were isolated from the intestine of wild oysters and perch, and a model of *Caenorhabditis elegans* infected by *Vibrio anguillarum* was established. Based on the model, ML1206, which showed a 99% similarity of 16S rRNA sequence to *Planococcus maritimus*, was selected as a potential marine probiotic, with strong antibacterial capabilities and great acid and bile salt tolerance, to protect *Caenorhabditis elegans* from being damaged by *Vibrio anguillarum*. Combined with plate counting and transmission electron microscopy, it was found that strain ML1206 could significantly inhibit *Vibrio anguillarum* colonization in the intestinal tract of *Caenorhabditis elegans*. Acute oral toxicity tests in mice showed that ML1206 was safe and non-toxic. The real-time qPCR results showed a higher expression level of genes related to the antibacterial peptide (*ilys-3*) and detoxification (*ugt-22*, *cyp-35A3*, and *cyp-14A3*) in the group of *Caenorhabditis elegans* protected by ML1206 compared to the control group. It is speculated that ML1206, as a potential probiotic, may inhibit the infection caused by *Vibrio anguillarum* through stimulating *Caenorhabditis elegans* to secrete antibacterial effectors and detoxification proteins. This paper provides a new direction for screening marine probiotics and an experimental basis to support the potential application of ML1206 as a marine probiotic in aquaculture.

## 1. Introduction

In recent years, with the development of the aquaculture industry and continuous expansion of intensive aquaculture, aquacultural diseases (such as bacterial diseases, fungal diseases, and viral diseases [1,2,3,4]) have increasingly been seen, which has caused substantial economic losses to the whole maricultural industry. To reduce the occurrence of aquacultural diseases, antibiotics have been widely used in the aquacultural industry for both preventive and therapeutic purposes. However, long-term usage of antibiotics will lead to the drug resistance of pathogens and environmental pollution [5].

Probiotics are widely considered as microorganisms with health benefits to the host, and are often used for disease control in aquaculture, especially for bacterial diseases. A diverse variety of Gram-positive and Gram-negative bacteria can be probiotics, including *Bacillus subtilis* [6], *Lactobacillus* spp. [7], *Pseudoalteromonas* spp. [8], etc. The various disease control mechanisms by probiotics include enhancement of immune response [9,10], competitive adhesion [11,12], pathogen antagonism [13,14], and disturbance of quorum sensing system [15].

Marine probiotics (such as *Pseudoalteromonas* sp. [16] and Marine actinobacteria [17]) are known to live in a particular environment of high pressure, high salt, low temperature, and low oligo nutrition. Thus, they have broad prospective applications in the aquacultural industry. At present, people mainly screen out marine probiotics from seawater, sediment, fish mucus, aquatic organism digestive tract, etc. [6,18,19,20].

*Vibrio anguillarum* (*V. anguillarum*), belonging to the genus *Vibrio*, family *Vibrionaceae*, can induce vibriosis under adverse conditions such as water pollution and high culture density. Vibriosis is one of the most severe bacterial diseases, with rapid infection and strong outbreak potential. The host species of *V. anguillarum* are numerous, including shrimp, crab, oyster, abalone, and other aquatic animals besides seawater fish. Symptoms of vibrio disease can vary depending on the host species. For example, for fish, symptoms include intestinal inflammation and bleeding, intestinal mucosa come-off, local congestion and even ulceration on the body surface [21,22]; for shrimps, symptoms include decreased food intake, weakened vitality, and a significant reduction in physical size [23].

*Caenorhabditis elegans* (*C. elegans*) is a tiny worm living in the soil. It consists of a digestive system, nervous system, muscle tissue, reproductive system, etc. From a scientific perspective, *C. elegans* shows the advantages of fast reproduction, easy observation and culture, full sequencing, uncomplicated infection, and so on. Therefore, it plays an irreplaceable role as a model organism in many research fields, such as antimicrobial drug discovery, probiotic mechanism, neurology, embryo development, and genetic variation [24]. 

So far, researchers have successfully established several infection models of *C. elegans*. Pathogenic bacteria that have been used to establish these models include *Staphylococcus aureus* [25], *Festuca faecalis* [26], *Listeria monocytogenes* [27]), *Vibrio cholerae* [28], *Salmonella* [29], *Enterotoxin Escherichia coli* [30], *Pseudomonas aeruginosa* [31], etc. However, screening marine probiotics against *Vibrio anguillarum* using *C. elegans* as a model organism has not been reported.

In this study, a model of *C. elegans* infected by *V. anguillarum* was established. ML1206, isolated from the intestinal tract of oysters, was screened from 20 different marine bacterial strains as a potential marine probiotic candidate using this model. The characteristics of ML1206 and its effects on the physiological performance of *C. elegans* were then analyzed. Based on a qRT-PCR analysis of *C. elegans*’s defense genes, we explored the genetic mechanisms via which ML1206 could protect *C. elegans* against *V. anguillarum* infection, such as the expression level of antibacterial and detoxification-related genes. Our study provides a new direction for further development and better utilization of marine probiotics in research and industry fields.

## 2. Results

### 2.1. Isolation and Identification of Non-Hemolytic Marine Bacteria from the Intestine of Oysters and Perch

A total of 41 marine bacterial strains were isolated and purified from the intestine of wild oysters (*Crassostrea gigas*) and perch (*Dictrarchus la Brax* L.) from Yuanyao dock in Weihai City, Shandong Province, China. 16S rRNA gene sequences of all the marine bacterial strains were obtained and compared to those available in the NCBI (www.ncbi.nlm.nih.gov/ (accessed on 21 February 2021)) nucleotide BLAST algorithm program. The similarity among different marine bacterial strains is listed in Table S1. To ensure there would be no hemolytic activity for probiotics upon acting on the host, which could lead to rupture of the host’s red blood cells, a hemolytic test was performed as a prerequisite, to screen and eliminate bacterial strains with active hemolytic activity. In this hemolytic test, *V. anguillarum* was used as a positive control [32]. Figure S1 shows the level of hemolytic activity for a few of the selected bacterial strains. For example, there was no obvious hemolytic ring around the colony of strain ML1206, indicating that it had no active hemolytic activity (Figure S1A). In contrast, an evident, clear, and transparent hemolytic ring around the colony edge of strain YLY14 indicates that it had active hemolytic activity (Figure S1B). In this way, the level of hemolytic activity was assessed for all 41 marine bacterial strains. The results show no hemolytic ring was observed for 20 out of 41 strains, suggesting that they did not produce hemolysin or possess any potential pathogenicity. The detailed results are presented in the supplementary Table S2. 

### 2.2. Detection of Acid and Bile Salt Tolerance by Marine Bacterial Strains

It is premised that probiotics can survive in the low pH and high bile salt environment inside the host’s gastrointestinal tract. In most in vitro tests, it is preferred to use pH 3.0 as the low pH to assess acid tolerance by different bacterial strains [33,34]. As shown in Table S3, the level of acid tolerance among different strains was significantly different. As a positive control, the survival rate of strain LGG was 118.71% ± 0.02%, while the survival rates of strain YLY25 and strain ML1206 were 85.24% ± 0.04%, and 79.51% ± 0.03%, respectively. Out of a total of 20 marine bacterial strains, six strains showed relatively good acid tolerance (survival rate > 50%). The order of acid tolerance was: YLY25 > ML1206 > YLY32 > YLY05 > YLY09 > YLY10 (Table S3).

For the bile salt tolerance tests, a bile salt concentration at 0.3% is considered the best condition [33,34]. In this experiment, the positive control LGG showed excellent bile salt tolerance. The survival rates of strain LGG, strain ML1206, and strain YLY06 were 87.42% ± 0.05%, 81.84% ± 0.04%, and 79.60% ± 0.03%, respectively (Table S4). There were five out of 20 marine bacterial strains that showed a good tolerance of bile salt (survival rate > 50%). The order of bile salt tolerance was: ML1206 > YLY06 > YLY05 > YLY09 > YLY02 (Table S4). 

Considering both the acid and bile salt tolerance tests, overall strain ML1206, strain YLY05, and strain YLY09 possessed good capabilities for both acid and bile salt tolerance.

### 2.3. Establishment of a C. elegans Infected by V. anguillarum Model

**Figure 1:**
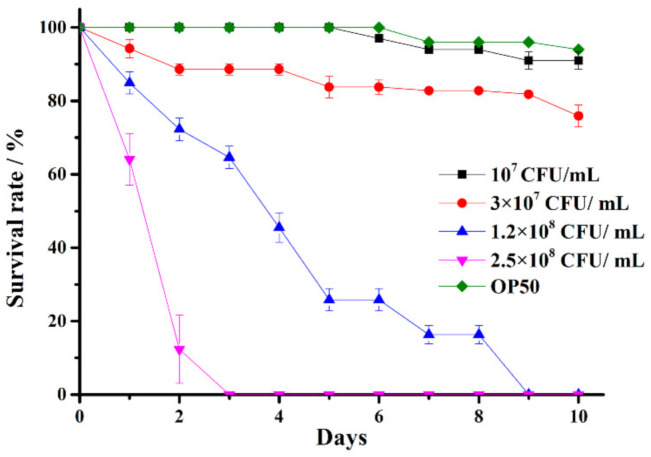
Establishment of *C. elegans* infected by *V. anguillarum* model. Note: *C. elegans glp-4* (*bn2*) worms were exposed to various concentrations of *V. anguillarum*. *C. elegans glp-4* (*bn2*) exposed to *E. coli* OP50 at a suspension concentration of 1.0 × 10^9^ CFU/mL were used as a control. There was a statistical significance with *p* < 0.05 in between the experimental groups (*C. elegans* infected by *V. anguillarum* at a concentration of 1.2 × 10^8^ CFU/mL and 2.5 × 10^8^ CFU/mL) and the control group. Results are presented in the form of mean ± SD of three independent experiments.

To establish a model in which *C. elegans* would die within 10 days of being infected by *V. anguillarum*, different concentrations of *V. anguillarum* were used to infect *C. elegans*. At *V. anguillarum* concentration of 1.0 × 10^7^ CFU/mL, the survival rate of *C. elegans glp-4* (*bn2*) on Day 10 was similar to that of the *Escherichia coli* (*E. coli*) OP50 control group (higher than 90%). When the concentration was increased to 3.0 × 10^7^ CFU/mL, the survival rate on Day 10 decreased by about 30%. When the concentration was further increased to 1.2 × 10^8^ CFU/mL, the *C. elegans glp-4* (*bn2*) all died on Day 9. Finally, at a concentration of 2.5 × 10^8^ CFU/mL, all the *C. elegans glp-4* (*bn2*) died on Day 3 (Figure 1).

These experimental results show that *V. anguillarum* was able to infect *C. elegans glp-4(bn2)* worms, and the death rate of *C. elegans glp-4(bn2)* was positively correlated to the concentration of pathogen *V. anguillarum*. When the concentration was above 1.0 × 10^7^ CFU/mL, the higher the concentration, the higher the death rate of *C. elegans glp-4(bn2)* on Day 10, and the shorter the survival duration. The final optimal concentration of *V. anguillarum* to kill *C. elegans glp-4(bn2)* within 10 days was set at 1.2 × 10^8^ CFU/mL. Therefore, the model of *C. elegans* infected by *V. anguillarum* was established, and was used to assess the capabilities of protecting *C. elegans* against *V. anguillarum* infection for several selected marine bacterial strains.

### 2.4. Screening of Potential Marine Probiotics Able to Protect C. elegans against V. anguillarum Infection

**Figure 2:**
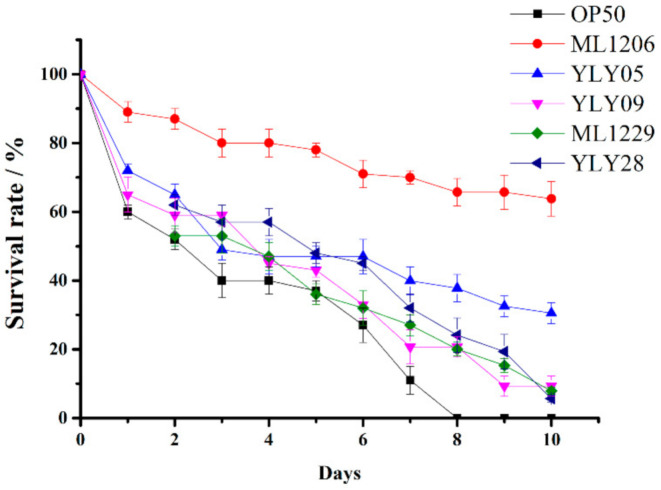
The survival rate of *C. elegans* against *V. anguillarum* infection under potential protection by marine bacterial strains. Note: *glp-4 (bn2) C. elegans* had been pre-cultured with either *E. coli* OP50 or a selected marine bacterial strain at a concentration of 1.0 × 10^9^ CFU/mL for 24 h before being exposed to *V. anguillarum* for 10 days. Results are presented in the form of mean ± SD of three independent experiments.

20 marine bacterial strains with no hemolytic activity were assessed for their ability to protect *C. elegans* from *V. anguillarum* infection in vivo. Table 1 shows that there was a significant difference in terms of in vivo pathogen-inhibiting ability among these strains. The survival rate of *C. elegans* varied from 0% to 63.57% on the last day (Day 10). Figure 2 shows the survival curve of *C. elegans* after being pre-cultured with *E. coli* OP50 or a selected marine bacterial strain at a concentration of 1.0 × 10^9^ CFU/mL for 24 h then being exposed to *V. anguillarum* for 10 days. Figure 2 includes the results for *E. coli* OP50 as the control and five experimental marine bacterial strains (ML1206, YLY05, YLY28, YLY09, and ML1229) with the highest *C. elegans* survival rate, with strain ML1206 and strain YLY05 having the highest survival rates of 63.57% and 30.75%, respectively. *C. elegans* in the control group had a 0% survival rate on Day 10 (Figure 2). 

By combining the results from the hemolysis test, acid and bile salt tolerance test, and in vivo anti-infection test, we found that the strain of *Planococcus maritimus* ML1206 presented the characteristics of no hemolysis-inducing ability, high level of acid and bile salt tolerance, and strong anti-infection capabilities against *V. anguillarum*. Therefore, ML1206 was considered to be the best marine probiotic candidate out of the 20 selected strains. Other characteristics of strain ML1206 and its underlying anti-infection mechanisms were further studied. The 16S rRNA gene sequence of ML1206 has been submitted to GenBank, and its accession number is MK332598.

### 2.5. Safety Evaluation of Strain ML1206

Antibiotic sensitivity was assessed for stain ML1206 according to the performance standards for antimicrobial susceptibility testing (PASAT) issued by the Clinical and Laboratory Standards Institute (CLSI). The testing results showed that ML1206 was susceptible to penicillin (10 µg), ofloxacin (5 µg), gentamycin (10 µg), chloramphenicol (30 µg), rifampin (5 µg), erythromycin (15 µg), cefotaxime sodium (30 µg), norfloxacin (30 µg), ampicillin (10 µg), clindamycin (30 µg), ceftriaxone (30 µg), carbenicillin (100 µg), kanamycin (30 µg), and neomycin (30 µg), but resistant to streptomycin (10 µg), tobramycin (10 µg), lincomycin (2 µg), and tetracycline (30 µg).

A toxicity test for strain ML1206 was performed according to the acute toxicity test standard of “National food safety standard: acute oral toxicity test, GB15193.3-2014, National Standard of the People’s Republic of China” [35]. There was no poisoning nor death observed for ICR mice (ICR mice is an albino strain, named by the Institute of Cancer Research in the USA) within 14-day exposure to ML1206, and the bodyweight of all male and female mice was normal. No abnormal changes were found in the gross anatomy of animals, and the acute toxicity dose (LD50) was larger than 10,017.6 mg/kg. According to the “acute toxicity test standards” [35], a LD50 value greater than 5000 mg/kg is considered non-toxic, thus ML1206 can be categorized into the non-toxic grade.

Based on the results of the antibiotic sensitivity test and toxicity test, it can be concluded that strain ML1206 is a food-grade, non-toxic, and safe candidate to be used as a potential marine probiotic.

### 2.6. Evaluation of ML1206 Presence on the Physiological Performance of C. elegans

**Figure 3:**
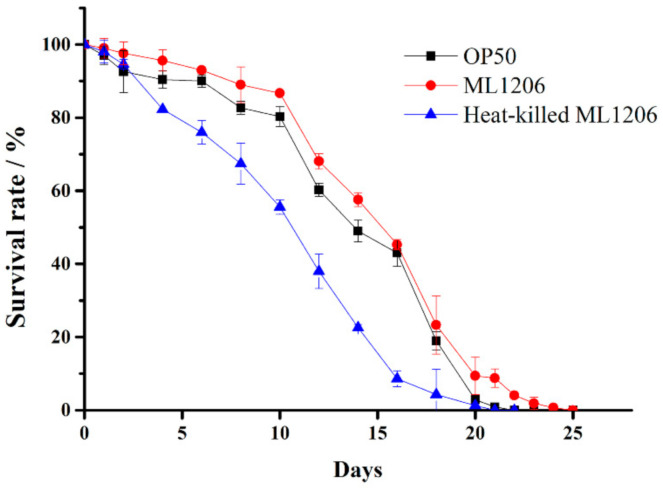
Influence of ML1206 on *C. elegans* (N2) life-span. Note: *C. elegans* N2 wild-type was fed with ML1206, heat-pretreated ML1206, and *E. coli* OP50, and the survival rate of *C. elegans* was recorded for a total duration of 25 days. All results are presented in the form of mean ± SD of three independent experiments.

To investigate whether strain ML1206, as a potential probiotic, would have a beneficial effect on maintaining the physiological performance of wild-type *C. elegans* (N2), the life span of *C. elegans* was studied. Figure 3 shows that the average life span of the control group fed with *E. coli* OP50 was 16.74 days, and there was no significant difference between the control group and one of the experimental groups fed with ML1206 that had been pre-treated with heat. However, compared to the control, the average life span of *C. elegans* was increased by 18.7% (*p* < 0.05) to 19.87 days for the other experimental group fed with normal ML1206, with the corresponding life curve shifted right. The results suggested that ML1206, as a potential probiotic, could prolong the life span of *C. elegans*. 

In addition, there was no significant difference observed in body length (Figure S2), body-bending (Figure S3), pharyngeal pump rate (Figure S4), and reproductive ability (Figure S5) between the experimental group fed with normal ML1206, and the control group fed with *E. coli* OP50, indicating that the presence of ML1206 had no adverse effects on the development, reproduction, and motility of *C. elegans*.

### 2.7. Assessment of ML1206 Inhibition on Intestinal Colonization of V. anguillarum in C. elegans

**Figure 4:**
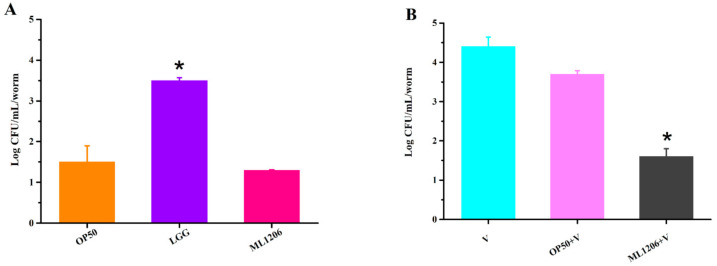
Plate counting of bacterial colonization in the intestine of *C. elegans.* Note: (**A**) Bacterial colonization in the intestines of *C. elegans* fed with *E. coli* OP50, LGG (*Lactobacillus rhamnosus*), and ML1206, respectively. (**B**) V stands for the group of *C. elegans* that were directly infected with *V. anguillarum*. OP50 + V and ML1206 + V represent the groups of *C. elegans* that had been pre-cultured with *E. coli* OP50 or ML1206 for 24 h first and then infected with *V. anguillarum,* respectively. Data is presented in the form of mean ± SD of three independent experiments. *: *p* < 0.05.

*C. elegans* is a well-known animal model for studying the host–bacteria interaction in intestines [36]. Colonization is generally considered as a prerequisite for following the growth and reproduction of probiotics in the host’s gut. *V. anguillarum* colonization in the intestinal tract of *C. elegans* was evaluated via plate counting and TEM in our study. Contrary to the previous expectation, ML1206 did not exhibit good colonization, and there was no statistical difference observed in terms of colonization level between the experimental ML1206 group (31 CFU/mL/*C. elegans*) and the negative control group of *E. coli* OP50 (20 CFU/mL/*C. elegans*) (Figure 4A). In contrast, the positive control group of LGG (*Lactobacillus rhamnosus*) (3.0 × 10^3^ CFU/mL/*C. elegans*) showed a significantly higher level of colonization in the intestinal environment [36], compared to ML1206 or *E. coli* OP50.

Figure 4B shows the plate counting results for assessing *V. anguillarum* colonization level in the intestines of *C. elegans*. The level of *V. anguillarum* colonization for the *C. elegans* pre-cultured with ML1206 (1.0 × 10^2^ CFU/mL/*C. elegans*) was significantly reduced compared to the group pre-cultured with *E. coli* OP50 (0.8 × 10^4^ CFU/mL/*C. elegans*) (*p* < 0.05), suggesting that ML1206 did not lead to a higher level of *V. anguillarum* colonization compared to *E. coli* OP50, but instead could inhibit colonization of *V. anguillarum* in the intestinal gut of *C. elegans*.

**Figure 5:**
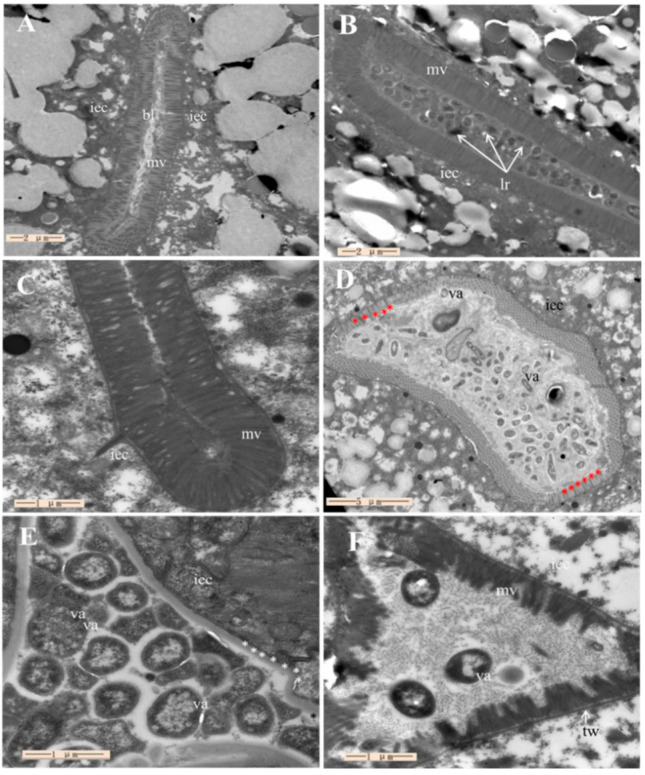
Electron micrographs of ML1206 and *V. anguillarum* colonization in the intestine of *C. elegans.* Note: (**A**) Group of *C. elegans* fed with *E. coli* OP50; bl: bowel lumen; mv: microvilli; iec: intestinal epithelial cells; (**B**) Group of *C. elegans* fed with LGG, lr: *L. rhamnosus* LGG; (**C**) Group of *C. elegans* fed with ML1206; (**D**) Group of *C. elegans* fed with *V. anguillarum*; (**E**) Group of *C. elegans* pre-cultured with *E. coli* OP50 for 24 h first, and then infected with *V. anguillarum*, va: *V. anguillarum*; (**F**) Group of *C. elegans* pre-cultured with ML1206 for 24 h first, and then infected with *V. anguillarum*. The red asterisks in Figure 5D represent the area where microvilli were slightly damaged or fell off. The white asterisks in Figure 5E represent the area where microvilli disappeared entirely. The scale is shown in the lower-left corner of each figure.

Following the above-mentioned colonization study, TEM was then used to visualize colonization and verify the previous colonization study results. Figure 5A shows no bacterial cell cluster in the intestine of *C. elegans* fed with *E. coli* OP50 (negative control), and the microvilli of the *C. elegans* were firm and dense, suggesting normal morphology of intestinal epithelial cells. Figure 5B shows the accumulation of LGG cells in the intestine of *C. elegans* (positive control). No visible bacterial cell clusters were found in the intestine after *C. elegans* were only fed with ML1206, as shown in Figure 5C, suggesting ML1206 could not colonize in the intestine of *C. elegans*. Figure 5D shows that *V. anguillarum* accumulated in the intestine in the form of cell clusters wrapped by the extracellular matrix. The microvilli of the *C. elegans* were either shorter than normal (red asterisks) or fell off. Figure 5E,F show the images of *C. elegans* that were first pre-cultured with *E. coli* OP50 (control) or ML1206, respectively, and were then infected with *V. anguillarum*. The images show that the intestinal tract of *C. elegans* pre-cultured with *E. coli* OP5 (Figure 5E) was significantly expanded, and its intestine was filled with a large number of *V. anguillarum*, while the level of *V. anguillarum* colonization in the group pre-cultured with ML1206 (Figure 5F) was significantly reduced.

The TEM images were consistent with the plate counting results shown in Figure 4. From both the TEM and plate counting studies, it was found that although ML1206 as a potential probiotic did not colonize the intestine of *C. elegans*, the level of intestinal colonization for *V. anguillarum* was significantly inhibited in the *C. elegans* that had been first pre-cultured with strain ML1206 before being exposed to *V. anguillarum*. Therefore, we hypothesize that the protection mechanism of ML1206 against *V. anguillarum* is through stimulating the immune response of *C. elegans* to *V. anguillarum* invasion.

### 2.8. Result of Real-Time Fluorescence Quantitative PCR

The twelve genes shown in Table 2 are known to be related to antibacterial effectors (C-type lectin, antibacterial peptide, and protease), the detoxification pathway (UDP-glucuronidase and cytochrome P450s), and the lipid metabolism pathway, and were therefore selected as candidate genes for qPCR verification, in order to study further the correlation between ML1206 and the immune response of *C. elegans.*

**Figure 6:**
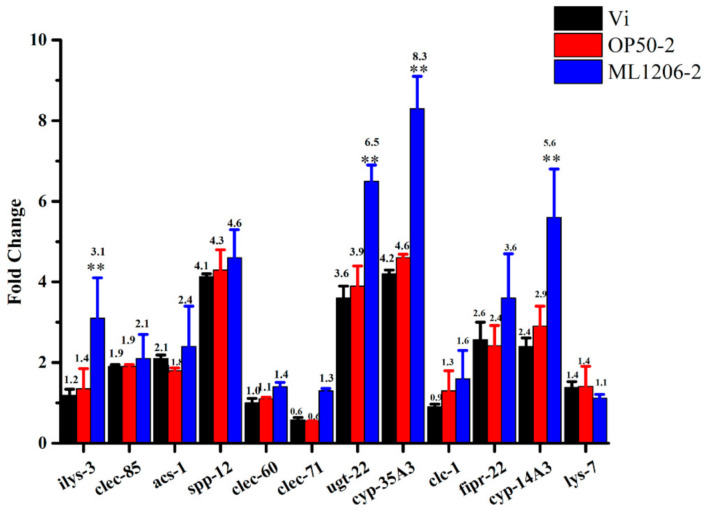
Level of transcription for different defense-related genes. Note: Vi: Group of *C. elegans* cultured with *V. anguillarum*; OP50-2: Group of *C. elegans* pre-cultured with *E. coli* OP50 for 24 h first, and then infected with *V. anguillarum*; ML1206-2: Group of *C. elegans* pre-cultured with *P. maritime* ML1206 for 24 h first, and then infected with *V.anguillarum*. The fold change for each gene was calculated with respect to the group of *C. elegans* cultured with *E. coli* OP50 (negative control). **: *p* < 0.05.

The analysis of qPCR results is shown in Figure 6. When *C. elegans* were cultured with *V. anguillarum*, the expression level of genes related to antimicrobial action (*clec-85*, *clec-60*, *clec-71, spp-12*, *fipr-22, clc-1*, *ilys-3,* and *lys-7*), detoxification (*ugt-22*, *cyp-35A3*, *cyp-14A3*), and lipid metabolism (*acs-1*) were up-regulated to different degrees. Especially, antibacterial gene *spp-12*, and detoxification genes *ugt-22* and *cyp-35A3* were up-regulated more than 3.6 times those in *C. elegans* cultured with *E. coli* OP50 (negative control). The general trend of expression level among different genes was similar between the *C. elegans* that had been pre-cultured with *E. coli* OP50 for 24 h first, and then infected with *V. anguillarum* (control), and the *C. elegans* that had been directly cultured with *V. anguillarum*.

After *C. elegans* were pre-cultured with *P. maritime* ML1206 for 24 h first, and then infected with *V. anguillarum*, the defense-related genes of the host were further stimulated, and especially the expression levels of antibacterial gene *ilys-3* and detoxification genes *ugt-22*, *cyp-35A3,* and *cyp-14A3* were all significantly increased, by about 2.2, 1.6, 1.8, and 1.9 times compared to the *E. coli* OP50 pre-cultured group (control), respectively. The results suggest that ML1206 might function as a potential probiotic via quickly stimulating a complex antibacterial innate immune response and further inducing detoxification activities in *C. elegans*, which would protect *C. elegans* from *V. anguillarum’s* infection, and even prolong the life span of *C. elegans*.

## 3. Discussion

In recent years, probiotics as microecological agents have been more widely used to enhance the immune function of aquatic animals, reduce the occurrence of diseases, purify water, and promote green and sustainable development in aquaculture [37]. Potential marine probiotics isolated from the intestines of healthy aquatic animals should have a advantage for survival in the intestinal environment of aquatic animals, and thus also promote their health [38,39,40,41]. *C. elegans* is considered a powerful model for studying the pathogenicity of microorganisms in vivo [24,25,28,42]. 

This paper is the first that has reported the successful establishment of a *C. elegans* model to measure its response to *V. anguillarum* infection. Using this *C. elegans* model, marine bacterial strains that may protect *C. elegans* against *V. anguillarum* infection can be screened and selected, and the underlying antibacterial mechanisms can be further studied. Several marine bacterial strains, which might inhibit *V. anguillarum* damage to *C. elegans*, were isolated from healthy intestines of oysters and perch, and were tested on the *C. elegans* model. The experiments of acid and bile salt tolerance are both considered indispensable steps in screening probiotics [43]. In this study, strain ML1206, with good tolerance of acid and bile salt, and antibacterial function, was selected out of 20 marine bacterial strains as a potential probiotic candidate for further experiments. 

An important way in which probiotics can protect the host against pathogenic bacteria is to reduce bacterial colonization in the host’s intestines and inhibit the following bacterial growth and reproduction, thus maintaining overall intestinal microbial balance inside the host [36]. In this study, we explored ML1206′s adhesion ability to intestinal epithelial cells of *C. elegans*, together with ML1206′s influence on the colonization level of *V. anguillarum* in the intestines of *C. elegans.* The results showed that, although ML1206 did not colonize the intestines of *C. elegans*, colonization of *V. anguillarum* was more inhibited in the intestines of *C. elegans* that had been pre-cultured with ML1206 compared to the group that had been pre-cultured with *E. coli* OP50. *Bacillus licheniformis,* a bacterial strain isolated from Korean traditional food sources, does not colonize the intestines of *C. elegans,* and has been proved to enhance the resistance of *C. elegans* to *Staphylococcus aureus* infection and also enhance the life span of *C. elegans* via mediating serotonin signaling [25,44]. Therefore, we speculated that ML1206 could protect *C. elegans* from being infected by *V. anguillarum* via inducing *C. elegans*’ immune response system.

Previous studies have shown that the main antibacterial genes of *C. elegans* function via stimulating the innate immune defense of *C. elegans* against pathogenic bacteria and fungi infection, which include the C-type lectin-like domain family (*clec-85*, *clec-60*, *clec-71*), Saposin-like protein family (*spp-12*), fungus-induced protein family (*fipr-22)*, Claudin-like in *Caenorhabditis* family (*clc-1*), and lysozyme (*ilys-3* and *lys-7*) [45,46]. In addition, many studies have reported that the UDP-glycosyltransferase family (*ugt-22*) and Cytochrome p450 family (*cyp-35A3* and *cyp-14A3)* are important detoxification-related genes, which can encode *C. elegans*’s natural detoxification immune proteins [47,48]. *Acs-1* gene of the Acyl-CoA synthetases family can regulate the skin’s lipid metabolism and barrier function to impact further the body’s immune defense system inside *C. elegans* [49,50,51]. 

The mRNA level of *ilys-3*, *ugt-22*, *cyp-35A3,* and *cyp-14A3* in the ML1206-pre-cultured *C. elegans* group was significantly higher than the *E. coli* OP50-pre-cultured *C. elegans* group, and the transcription level was increased 2.2, 1.6, 1.8, and 1.9 times, respectively. A previous study showed that lysozymes coded by *ilys-3* gene could disrupt bacteria under non-pathogenic growth conditions while being expressed constitutively in pharyxes of *C. elegans*, and that its lytic activity could break the cell-wall of bacterial pathogens such as *Microbacterium nematophilum* [52,53]. The study further suggested that the *ilys-3* gene could be strongly up-regulated in intestinal cells of *C. elegans* after starvation or exposure to *Microbacterium nematophilum*, thus protecting intestines from bacterial pathogenic damage. Referring to those results, we speculated that ML1206 might antagonize against *V. anguillarum* and inhibit the colonization of *V. anguillarum* by inducing the mRNA transcription of the gene *ilys-3* inside *C. elegans*. Furthermore, the intestine of *C. elegans* is known as a detoxification organ, in which proteins encoded by detoxification-related genes play a physiological role [47,54]. ML1206 was also likely to reduce *V. anguillarum*’s toxicological impact by inducing the expression of detoxification genes *ugt-22*, *cyp-35A3,* and *cyp-14A3,* so as to protect *C. elegans* against *V. anguillarum* infection.

In conclusion, in this study a *C. elegans* model was established to screen marine bacterial strains that could protect *C. elegans* against *V. anguillarum* infection and potentially be used as marine probiotics. *P. maritimus* ML1206, with good tolerance of acid and bile salt and antibacterial capability in vivo, was selected out of a total of 20 strains, and its antibacterial mechanisms against *V. anguillarum* infection were studied. It was speculated that ML1206 could induce the innate immune response inside *C. elegans* and stimulate *C. elegans* to secrete many antibacterial effectors and detoxification proteins, thus significantly reducing the pathogen’s damage to *C. elegans*. The results presented in this study will provide a new method for screening marine probiotics, a new direction for further studying ML1206′s antibacterial mechanisms, and an experimental basis to support the potential use of ML1206 as a marine probiotic in aquaculture.

## 4. Materials and Methods

### 4.1. Strains and Growth Conditions

The intestinal bacteria used in this study had been isolated from the intestines of wild oysters (*Crassostrea gigas*) and perch (*Dictrarchus la Brax l.*) from the Yuanyao dock in Weihai City, Shandong Province, China. The intestinal tissues were detached, homogenized, and serially diluted to 10^−4^ with sterilized seawater, and 100 μL aliquots of each dilution were spread onto marine agar 2216 (MA; Becton Dickinson, Franklin Lakes, NJ, USA), respectively [55]. Each sample was cultured in an incubator at 28 °C for 7 days. *Vibrio anguillarum* (*V. anguillarum)*, SDUM002245, was obtained from the Strain Collection Center at Shandong University in Weihai, and was cultured on MA at 28 °C. *Escherichia coli* (*E. coli*, OP50), standard food for *C. elegans*, was grown in Luria-Bertani (LB) broth at 37 °C [56]. *Lactobacillus rhamnosus* (LGG), one of the most widely recognized probiotics, is known to colonize the intestinal tract of *C. elegans* and have good tolerance of acid and bile salt [36]. LGG was purchased from Culturelle^®^ (distributed by I-health, Inc., Cromwell, CT, USA) and was cultured in De Man, Rogosa, and Sharpe (MRS) broth at 37 °C. 

Two *C. elegans* strains were obtained from the Caenorhabditis Genetics Center (CGC, University of Minnesota, MN, MINNEAPOLIS, USA) and were maintained at 20 °C in *C. elegans* growth media (NGM) seeded with *E. coli* OP50. *C. elegan* strains used in this study were N2 Bristol (wild type) and *glp-4 (bn2)* [56]. *glp-4 (bn2)* does not reproduce at 25 °C, as its reproduction is temperature-sensitive. 

To prepare live bacteria lawns for *C. elegans* feeding, *E. coli* OP50 or an experimental bacterial strain was harvested and washed three times in M9 buffer (Na_2_HPO_4_ 6 g, KH_2_PO_4_ 3 g, NaCl 5 g, MgSO_4_7H_2_O 0.25 g in 1 L of distilled water and autoclaving), then was adjusted to a final concentration of 1.0 mg per μL in M9 buffer and seeded on NGM (NaCl 3 g, Bacto peptone 2.5 g, 1 M potassium phosphate (pH 6.0) 25 mL, agar 17 g in 1 L of distilled water and autoclaving, then added cholesterol (5 mg/mL) 1 mL, 1 M CaCl_2_ 1 mL and 1 M MgSO_4_ 1 mL) plate mentioned above [56].

### 4.2. 16S rRNA Gene Sequencing and Analysis

Extraction of genomic DNA was performed by using a DNA kit or the boiling method. The DNA kit method was performed by following the protocol provided by the manufacturer (TaKaRa Bio, Kusatsu, Japan). The boiling method was to suspend a single colony pellet in 30 μL of double-distilled water, which was subjected to boiling in a hot water bath at 100 °C for 10 min, followed by cooling in a freezer at −20 °C for 5 min so that bacteria could be fully lysed. Then, 1 μL of the bacterial lysate was used as a DNA template [57]. 16S rRNA gene of different bacterial strains was amplified from corresponding genomic DNA by PCR using two universal primers, 27 F and 1492 R [58]. The reaction was performed in a total volume of 50 μL mixture, composed of 25 μL 2×Taq plus PCR Master Mix (RuiBiotech, Beijing, China), 1 μL of each primer (20 μM), 1 μL of DNA template, and 22 μL of MiliQ water. PCR amplification condition was one cycle at 94 °C for 3 min, then thirty cycles at 94 °C for 45 s, 53 °C for 30 s, and 72 °C for 90 s, and finally one cycle at 72 °C for 10 min. PCR products were purified using a MicroElute DNA Clean-Up Kit (OMEGA, Guangzhou, China). The purified PCR products were ligated into a pGM-T vector (Tiangen, Beijing, China) for cloning, as described by Liu et al. [59]. Sequencing was performed by Life Biotechnology (Shanghai, China), and sequence similarities were obtained via the NCBI (www.ncbi.nlm.nih.gov/ (accessed on 26 February 2021)) nucleotide BLAST algorithm program [60]. A near-complete sequence (1452 bp) of ML1206 has been submitted to GenBank, and its accession number is MK332598.

### 4.3. Hemolysis Experiment

Hemolysis assays were performed on blood agar plates containing 5% sheep blood and 1% agar. A colony of each bacterial strain was inoculated on a blood agar plate. Three parallel plates were set up for each bacterial strain. The hemolysis level was evaluated according to the number of transparent hemolytic rings produced [61]. 

### 4.4. Experiment of Acid and Bile Salt Tolerance

The experiments of acid and bile salt tolerance were performed to screen marine probiotics that could potentially survive in the intestinal environment of *C. elegans*. The acid tolerance test was conducted by following the method described by Tulumoglu et al. [62], with some modifications. The experimental group of different marine bacterial strains and the positive control strain (LGG) were cultured overnight in MA (pH 7.4) and MRS (pH 5.6) liquid medium, respectively. Then, 1.5 mL of bacterial suspension was centrifuged at 6500 rpm speed for 5 min, and the supernatant was discarded. The collected bacteria were washed three times with PBS buffer at pH 7.4 (marine strain) or pH 5.6 (LGG). After incubation in PBS of 1.5 mL at pH 3.0 (marine strain or LGG) and pH 7.4 (marine strain) or pH 5.6 (LGG) for 2 h, 250 μL of bacterial PBS suspension was inoculated into MA or MRS liquid medium to 5% at the end, respectively. After 24 h of culture, the OD value of different strains at 600 nm wavelength was determined. The survival rate of each strain at different pH was calculated according to the formula: survival rate (%) = (A_2_ − A_0_)/(A_1_ − A_0_) × 100% (A_2_ is OD_600_ of the culture medium at pH 3.0; A_1_ is OD_600_ of the culture medium at pH 7.4 for the marine bacterial strain, and pH 5.6 for LGG; A_0_ is OD_600_ of the blank control).

The bile salt tolerance test was conducted by following the experimental method described by Arihara et al. [63], with some modifications. For all the steps before incubation in PBS, each experimental bacterial strain or LGG (positive control) was prepared in the same way as in the above-mentioned acid tolerance test. For incubation, each strain from the experimental group or LGG was incubated in 1.5 mL PBS at pH 7.4 (marine strain) or pH 5.6 (LGG) for 2 h. Then, 250 μL of bacterial PBS suspension was inoculated into a MA or MRS liquid medium to 5% at the end, with a bile salt concentration of either 0% or 0.3%, and was cultured at 28 °C for 24 h. The survival rate of each strain was calculated according to the formula: survival rate (%) = (A_2_ − A_0_)/(A_1_ − A_0_) × 100% (A_2_ is OD_600_ value of the medium containing 0.3% bile salt; A_1_ is OD_600_ value of the medium with 0% bile salt; A_0_ is OD_600_ value of the blank control).

### 4.5. Establishment of the Model of C. elegans Infected with V. anguillarum

The synchronized *glp-4 (bn2)* mutants in the L4 stage were collected and washed three times using M9 buffer. For the experimental group, a 2 mL aliquot of S culture solution (NaCl 5.9 g, 1 M potassium phosphate 25 mL NaCl 3 g, cholesterol (5 mg/mL) 1 mL, 1 M CaCl_2_ 1 mL, 1 M MgSO_4_ 1 mL in 1 L of distilled water and autoclaving) containing different concentrations of *V. anguillarum* (1.0 × 10^7^ CFU/mL, 3.0 × 10^7^ CFU/mL, 1.2 × 10^8^ CFU/mL, and 2.5 × 10^8^ CFU/mL) was respectively added to a 24-well plate well seeded with 15–20 *C. elegans*. In the control group, a 2 mL aliquot of S culture solution containing 1.0 × 10^9^ CFU/mL *E. coli* OP50 was added to a well seeded with 15–20 *C. elegans*. For each experimental or control group, three independent wells were performed. Then the 24-well plate was stored at 25 °C for 10 days. The living status of *C. elegans* was observed under stereomicroscope and recorded daily. A *C. elegans* worm was considered alive when it was swinging or responded to a gentle touch by a platinum wire pick, while a worm was considered dead when it failed to respond to a gentle touch by a platinum wire pick. The survival rate at each *V. anguillarum* or *E. coli* OP50 concentration was calculated using the formula: survival rate (%) = number of surviving worms/number of total worms × 100% [64]. The concentration of *V. anguillarum* with 0% survival rate of *C. elegans* on the tenth day was used as the final concentration for the infection model.

### 4.6. Screening of Potential Marine Probiotics against V. anguillarum Infection Using the C. elegans Model

First, synchronized *C. elegans glp-4 (bn2)* mutants in the L4 stage were transferred to a 2 mL aliquot of S culture solution in a 24-well plate well, and a different marine bacterial strain at 1.0 × 10^8^ CFU/mL (experimental group) or *E. coli* OP50 at 1.0 × 10^9^ CFU/mL (control group) was added to each well and cultured at 25 °C for 24 h. This first step was considered as the pre-treatment for *C. elegans glp-4 (bn2)* mutants. Second, the pretreated *C. elegans* were washed three times with M9, respectively. Finally, 15–20 of *C. elegans* from each strain of the experimental group or the control group were transferred to a 24-well plate well containing 2 mL of S culture solution and *V. anguillarum* at 1.2 × 10^8^ CFU/mL, and were cultured at 25 °C for 10 days.

The living status of *C. elegans* was observed under stereomicroscope and recorded daily. A *C. elegans* was considered dead when it failed to respond to a gentle touch by a platinum wire pick. The survival rate of *C. elegans* was calculated using the same approach as described in Section 4.5 [30].

### 4.7. Antibiotic Susceptibility Assay

Antibiotic susceptibility assay was performed on MA using the disc diffusion method according to Du et al. [65]: the first step was that a bacterial suspension (McFarland standard 0.5) was swabbed on the surface of MA to produce a uniform lawn; the second step involved aseptic placement of antibiotic discs onto the surface of the MA at 28 °C for 48 h. Antibiotic susceptibility was determined by measuring the diameter of each antibacterial circle with a ruler. For each strain, the test was repeated three times.

### 4.8. Safety Test of ML1206

The freeze-dried ML1206 was sent to the Inspection and Quarantine Technical Center of Ningbo Entry-Exit Inspection and Quarantine Bureau for acute oral toxicity testing. The test was performed as follows: 20 ICR mice were prepared (provided by Zhejiang Experimental Animal Center, Zhejiang, China) with an average weight of 20 g and half male and half female. According to the maximum limit method, the mice were exposed to oral poisoning twice a day by oral gavage. The daily intragastric dose was 0.6 mg/kg, and each mouse was fasted before and after exposure. The living status of ICR mice was observed daily, and the number of poisoned and dead mice was recorded over a total duration of 28 days. Once the experiment was completed, any remaining alive ICR mice were sacrificed and grossly dissected [66].

### 4.9. C. elegans Life Span Assay

The synchronized *C. elegans* N2 wild-type in the L4 stage were transferred to 60 mm diameter Petri dishes that contained 5 mL NGM (0.1 mg/mL FUDR) per dish, and each dish was seeded with *E. coli* OP50, active ML1206, or inactivated ML1206 (incubated in a water bath at 80 °C for 30 min). Next, 60 *C. elegans* were divided evenly into three Petri dishes for each *E. coli* OP50 or ML1206 group and cultured at 20 °C. The number of alive *C. elegans* was counted every two days. A *C. elegans* worm was considered dead when it failed to respond to a gentle touch by a platinum wire pick. *C. elegans* in the following scenarios were not counted: (1) climbed onto the wall of the Petri dish; (2) accidentally died while the assay was being performed (such as died after skin breaking, climbed onto the wall of the Petri dish and died). For each group, the life span assay was repeated three times [67].

### 4.10. Plate Counting for Intestinal Colonization of V. anguillarum

The synchronized *C. elegans glp-4 (bn2)* mutants in the L4 stage were transferred to 60 mm Petri dishes that contained 5 mL NGM per dish, and each dish was seeded with a different bacterial strain. *C. elegans* were divided into a total of 6 groups: (1) Group cultured with *E. coli* OP50 (negative control); (2) Group cultured with LGG (positive control: it is only used to judge intestinal colonization); (3) Group cultured with ML1206; (4) Group cultured with *V. anguillarum*; (5) Group cultured with *E. coli* OP50 for 24 h, then with *V. anguillarum* added; (6) Group cultured with ML1206 for 24 h, then with *V. anguillarum* added.

Subsequently, 10 *C. elegans* in the 3-day adult stage were picked randomly from each group, washed three times with M9 buffer, and soaked in 70% alcohol for 20 s to disinfect the body surface of bacteria. After that, *C. elegans* were washed three times using M9 buffer. The washed worms were lysed in M9 buffer with 1% triton X-10 and crushed using a sterile grinding rod. The lysates were serially diluted in M9 buffer, spread onto the plates and incubated overnight. For *E. coli* OP50, LB plates were used at 37 °C; for LGG, MRS plates were used at 37 °C; for ML1206 or *V. anguillarum*, MA plates were used at 28 °C; for the *E. coli* OP50-pre-cultured group and ML1206-pre-cultured group, MA plates containing 25 μg/mL gentamicin and 100 μg/mL clindamycin were used at 28 °C. Colonies were counted and used to reflect the number of bacteria colonizing intestinally inside *C. elegans* [30,44,68].

### 4.11. Transmission Electron Microscope Observation

Using the same grouping as in Section 4.10, all collected *C. elegans* were washed three times with M9 buffer, then fixed with a 2.5% dialdehyde solution and sent to the Electron Microscope Center at Qingdao University for TEM observation. The fixed *C. elegans* were dehydrated, embedded, and solidified using a Reichert Jung ultrathin section machine (Reichert-Jung, Vienna, Austria), 70 nm slice, and copper mesh. The ultrathin sections were stained with uranium acetate and lead citrate for 15 min. At least ten cross-sections were evaluated for each observation under TEM, and representative images were photographed under a JEM 1200 electron microscope (JEOL, Tokyo, Japan) [69].

### 4.12. Real-time Fluorescence Quantitative PCR Analysis

Two thousand synchronized *C. elegans glp-4 (bn2)* worms in the 3-day adult stage from four groups were collected: (1) Group cultured with *E. coli* OP50 (negative control; (2) Group cultured with *V. anguillarum*; (3) Group cultured with *E. coli* OP50 for 24 h, then with *V. anguillarum* added (control); (4) Group cultured with ML1206 for 24 h, then with *V. anguillarum* added. RNA from the *C. elegans* mentioned above were then isolated with an Illumina Truseq TMRNA spin Mini RNA Isolation Kit (GE Healthcare, Buckinghamshire HP7 9NA, UK). For each group, a total of 300 ng RNA was reverse transcribed using an ABscript II cDNA First-Strand Synthesis Kit (ABclonal, Wuhan, China). The sequences of target genes were then found from www.wormbase.org (accessed on 21 February 2021), and qPCR primers for each gene were subsequently designed by Primer3Plus (Copyright (c) 2006, 2007 by Andreas Untergasser and Harm Nijveen) [67]. In this study, *act-1* was used as an internal reference to evaluate the relative qPCR expression among different genes [70]. Primers for each target gene were tested for their efficiency using a dilution series of the cDNA template, and the information of primers is listed in Table 2. The qPCR reaction was performed using an ABI StepOnePlus thermal cycler under the following condition: 95 °C for 2 min, then 40 cycles of 95 °C for 15 s, 60 °C for 30 s, and 72 °C for 30 s. The qPCR reaction was performed in a total volume of 20 μL, composed of 10 μL 2X Universal SYBR Green Fast qPCR Mix (ABclonal, Wuhan, China), 0.4 μL of each primer (10 μM), 0.4 μL of a sample, and 8.8 μL of MiliQ water. Negative assay controls included both a no-template cDNA control and a no-reverse transcriptase control. qPCR relative expression level was calculated using the 2-△△Ct method [58]. The experiment was repeated three times.

### 4.13. Statistical Analysis

All experiments were performed at least in triplicate. Statistical analysis was performed using origin9.0 software (OriginLab, Northampton, USA). The results were reported in the form of mean ± standard deviation (SD) and analyzed using one-way ANOVA and Student’s test. Differences with *p* values < 0.05 were considered significant. All bacterial infection and qPCR studies were repeated in at least three independent experiments with comparable results obtained. Data from a representative set of experiments were presented in this paper.

## Figures and Tables

**Table 1:** Survival Rate of *C. elegans* on Day 10 after Exposure to Different Marine Bacterial Strains for 24 h Followed by *V. anguillarum* Infection (Average ± Standard Deviation).

Strain Number	Source ^#^	Putative Identity	Survival Rate (%)
M0102	C	*Marinobacter hydrocarbonoclasticus 98.8%*	0.00 ^k^
ML1206	C	*Planococcus maritimus 99%*	63.57 ± 0.79 ^a^
ML1210	C	*Sulfitobacter pontiacus 98.3%*	11.32 ± 0.35 ^g^
ML1229	C	*Ruegeria atlantica 99.3%*	20.02 ± 0.35 ^d^
YLY02	D	*Vibrio alfacsensis 99.5%*	0.00 ^k^
YLY03	D	*Sunxiuqinia elliptica 99.1%*	4.35 ± 0.29 ^h^
YLY04	D	*Maribius pontilimi 95.33%*	12.37 ± 0.33 ^f^
YLY05	D	*Idiomarina sediminum 98.9%*	30.75 ± 0.34 ^b^
YLY06	D	*Vibrio orientalis 100%*	1.56 ± 0.11 ^j^
YLY07	D	*Brumimicrobium mesophilum 95.2%*	0.00 ^k^
YLY08	D	*Oceaniglobus indicus 93.4%*	0.00 ^k^
YLY09	D	*Nitratireductor aquimarinus 98.1%*	20.18 ± 0.25 ^d^
YLY10	D	*Microbacterium esteraromaticum 98.1%*	1.47 ± 0.23 ^j^
YLY20	D	*Ornithinimicrobium kibberense 95.7%*	0.00 ^k^
YLY21	D	*Litoreibacter arenae 99.8%*	1.98 ± 0.32 ^j^
YLY25	D	*Marinobacter pelagius 98.9%*	0.00 ^k^
YLY26	D	*Stappia stellulata 99.1%*	0.00 ^k^
YLY27	D	*Idiomarina aestuarii 99.4%*	3.54 ± 0.27 ^i^
YLY28	D	*Roseovarius halotolerans 98.9%*	22.45 ± 0.72 ^c^
YLY32	D	*Halomonas aestuarii 96.8%*	0.00 ^k^
*E. coli* OP50			0.00 ^k^

Note: Different lowercase letters in superscript represent the significant difference between every two strains (*p* < 0.05); ^#^: C, isolated from the oyster intestine; D, isolated from perch intestine.

**Table 2:** Defense Related Genes of *C. elegans* and Primer for qPCR.

Gene Name	Accession Number	Size (bp)	Description	Forward_Seq (5′ to 3′)	Reverse_Seq (5′ to 3′)	Efficiency (%)
act-1 *	NM_073418.9	121	Actin-1	CCCCACTCAATCCAAAGGCT	GTACGTCCGGAAGCGTAGAG	100.0
*ilys-3*	NM_067805.5	194	Invertebrate-type lysozyme 3	CCGGAGAAACAACTGAAGCC	TGTGGTTACGAGCCATCACT	101.0
*clec-85*	NM_001383060.2	121	C-type LECtin	CCAATGGGATGACGGAACCA	CTTCTGTCCAGCCAACGTCT	103.4
*acs-1*	NM_001392534.1	186	Fatty Acid CoA Synthetase family	CTTATTCGCAAGTCGCCACA	CAAGAGCACTGGCAAACTGT	100.2
*spp-12*	NM_074042.3	123	SaPosin-like Protein family	AGGAAGCTGGAGATGTTGCT	AGATGTCATGCTCAGCCACT	104
*clec-60*	NM_063858.4	199	C-type LECtin	TGGTGGACAACTCGATTGGA	CCGCAGCTTTGTTGTAGGTT	99.8
*clec-71*	NM_068039.4	227	C-type LECtin	TTGGCTGTTGTAGGCAATCAA	TCACTGGGAATCCGTTATCC	99.9
*ugt-22*	NM_070232.6	168	UDP-glucuronosyltransferase	ACATGTTGAAACGGCATGGT	TGTCCTTTGGATTCGTTGGC	102.5
*cyp-35A3*	NM_071720.6	185	Cytochrome P450 family	GGGCCTATTTCCTTCCCACT	CGGAACTGGACCAACCCATA	100.9
*clc-1*	NM_077446.6	175	CLaudin-like in Caenorhabditis	TGAAATGTGTCGTCGTCTGC	TGCAAAGAGGGTGAGTGGAT	98.1
*cyp-14A3*	NM_077804.4	133	Chrome P450 family	TTGCGTTGGTGAAGGATTGG	TGGCTTTGTTGTCAGAACCG	101.6
*lys-7*	NM_071571.9	153	Lysozyme-like protein 7	GTACAGCGGTGGAGTCACTG	GCCTTGAGCACATTTCCAGC	96.6
*fipr-22*	NM_061069.5	184	Fungus-Induced Protein Related Cytochrome P450 family	ATCGGAGCATTCTGTGCAAC	CATACTGGATTGGGCTTCCG	105.0

* *act-1* is an internal reference gene.

## Data Availability

The data presented in this study are available in the main text and the supplementary materials of this article.

## Supplementary Materials

The following are available online at https://www.mdpi.com/1660-3397/19/3/150/s1, Figure S1: Hemolysis of some strains on 5% sheep blood agar (partial), Figure S2: Effect of ML1206 strain on *C. elegans* growth, Figure S3: Effect of ML1206 strain on *C. elegans* body bending frequency, Figure S4: Effect of ML1206 strain on *C. elegans* pharyngeal pumping rate, Figure S5: Effect of ML1206 strain on *C. elegans* reproduction ability, Table S1: Stains from the intestine of oyster and perch, Table S2. Hemolysis of strains, Table S3: Low pH tolerance ability of twenty intestinal bacteria, Table S4. Bile salt tolerance ability of twenty intestinal bacteria.
