# Supplementary material for: Planococcus maritimus ML1206 Isolated from Wild Oysters Enhances the Survival of Caenorhabditis elegans against Vibrio anguillarum
Source: Mar Drugs. 2021 Mar 12;19(3):150. doi: 10.3390/md19030150 (PMC7999227; doi:10.3390/md19030150)
Supplement: Supplementary file 1 [file marinedrugs-19-00150-s001.pdf]

# *Planococcus maritimus* ML1206 Isolated from Wild Oysters Enhances the Survival of *Caenorhabditis elegans* against *Vibrio anguillarum*

**Ying-Xiu Li [1], Nan-Nan Wang [1], Yan-Xia Zhou [1], Chun-Guo Lin [1], Jing-Shan Wu [1], Xin-Qi Chen [1], Guan-Jun Chen [1,2,*] and Zong-Jun Du [1,2,*]**

[1] Marine College, Shandong University, Weihai 264209, China; yingxiuli@sdu.edu.cn (Y.L.); wangnan2016@mail.sdu.edu.cn (N.W.); zhouyx@sdu.edu.cn (Y.Z.); linchunguo@mail.sdu.edu.cn (C.L.); wujingshan@mail.sdu.edu.cn (J.W.); chenxinqi@mail.sdu.edu.cn (X.C.)
[2] State Key Laboratory of Microbial Technology, Shandong University, Jinan 250100, China
* Correspondence: guanjun@sdu.edu.cn (G.C.); duzongjun@sdu.edu.cn (Z.D.)

## Method

### 1. *C. elegans* Length Determination

Ten synchronized *C. elegans* N2 in L1 stage were transferred to NGM medium coated with OP50 and ML1206, respectively. *C. elegans* were cultured at 20 °C to the 2-days adult stage. *C. elegans* were picked to slides covered with agarose, anesthetized with levamisole hydrochloride, and placed under a stereomicroscope to measure body length with a micrometer. The head (oral position) of the *C. elegans* was taken as the starting point, and following the middle part of the body to the tail part of the *C. elegans* was taken as the measurement endpoint. NIS elements software was used to calculate the body of the *C. elegans* body [1]. The test was performed three times.

### 2. *C. elegans* Motility Determination

Referring to the methods of Tsalik and Hobert[2], the effect of ML1206 on *C. elegans* motility was tested. Ten synchronized *C. elegans* N2 in L1 stage were transferred to NGM medium coated with OP50 and ML1206, respectively. *C. elegans* were cultured at 20 °C to the 2-days adult stage. Then the *C. elegans* were transferred to new NGM plates. After the free movement of *C. elegans* in the first minute, the bending times of the *C. elegans* within 30 s were observed and recorded under a stereoscopic microscope as the indicator of bending frequency. One bending of the body is defined as a change in the movement of the body along the corresponding X-axis during *C. elegans* crawling, assuming that the direction along the pharyngeal pump is the Y-axis[3]. The test was performed three times.

### 3. *C. elegans* Pharyngeal Pumping Experiment

Ten synchronized *C. elegans* N2 in L1 stage were transferred to NGM medium coated with OP50 and ML1206, respectively. *C. elegans* were cultured at 20 °C to the 2-days adult stage. Then the *C. elegans* were transferred to new NGM plates, the pump activity of *C. elegans* pharyngeal terminal bulb was observed through stereomicroscope (×400), and the number of pharyngeal pump activities within 1 min was recorded, the swallowing rate of *C. elegans* [1]. The test was performed three times.

### 4. *C. elegans* Reproductive Capacity Determination

Ten synchronized L1 N2 were selected to NGM medium coated with OP50 and ML1206, respectively. One plate was for one worm and kept *C. elegans* lay eggs at 20 °C. The parent *C. elegans* were transferred to a new NGM medium coated with OP50 and ML1206 every 12 h, and the number of eggs in each old culture dish was recorded until the *C. elegans* stopped laying eggs. The sum of eggs in plates of each group was the total number of eggs laid by 10 *C. elegans*, and the averages of offspring produced by each *C. elegans* were calculated to characterize the reproductive capacity of the parent *C. elegans*[1]. The test was performed three times.

## Results

Table Sl. Stains from the intestine of oyster and perch

| number | Stain number | Putative identity |
|---|---|---|
| 1 | M0101 | *Pseudoalteromonas haloplanktis* 98.2% |

| | | |
|---|---|---|
| 2 | M0102 | *Marinobacter hydrocarbonoclasticus* 98.8% |
| 3 | M0103 | *Psychrobacter celer* 98.8% |
| 4 | M0104 | *Pseudoalteromonas nigrifaciens* 98.1% |
| 5 | M0106 | *Lacinutrix undariae* 98.0% |
| 6 | M0107 | *Pseudoalteromonas translucida* 98.2% |
| 7 | M1201 | *Bacillus siamensis* 98.0% |
| 8 | ML1202 | *Bacillus tequilensis* 98.9% |
| 9 | ML1206 | *Planococcus maritimus* 99.2% |
| 10 | ML1209 | *Pseudoalteromonas fuliginea* 99.2% |
| 11 | ML1210 | *Sulfitobacter pontiacus* 98.3% |
| 12 | ML1211 | *Olleya algicola* 99.9% |
| 13 | ML1212 | *Bacillus flexus* 98.8% |
| 14 | ML1216 | *Bacillus firmus* 97.5% |
| 15 | ML1224 | *Pseudoalteromonas neustonica* 97.3% |
| 16 | ML1226 | *Pseudovibrio japonicus* 99.0% |
| 17 | ML1227 | *Bacillus altitudinis* 98.4% |
| 18 | ML1229 | *Ruegeria atlantica* 99.3% |
| 19 | YLY02 | *Vibrio alfacsensis* 99.5% |
| 20 | YLY03 | *Sunxiuqinia elliptica* 99.1% |
| 21 | YLY04 | Maribius pontilimi 95.33% |
| 22 | YLY05 | *Idiomarina sediminum* 98.9% |
| 23 | YLY06 | *Vibrio orientalis* 100% |
| 24 | YLY07 | *Brumimicrobium mesophilum* 95.2% |
| 25 | YLY08 | *Oceaniglobus indicus* 93.4% |
| 26 | YLY09 | *Nitratireductor aquimarinus* 98.1% |
| 27 | YLY10 | *Microbacterium esteraromaticum* 98.1% |
| 28 | YLY13 | *Bacillus halosaccharovorans* 98.8% |
| 29 | YLY14 | *Roseovarius pacificus* 99.6% |
| 30 | YLY17 | *Shewanella indica* 98.6% |
| 31 | YLY18 | *Vibrio atypicus* 99.9% |
| 32 | YLY20 | *Ornithinimicrobium kibberense* 95.7% |
| 33 | YLY21 | *Litoreibacter arenae* 99.8% |
| 34 | YLY22 | *Halobacillus alkaliphilus* 97.8% |
| 35 | YLY23 | *Jeotgalibacillus campisalis* 99.0% |
| 36 | YLY25 | *Marinobacter pelagius* 98.9% |
| 37 | YLY26 | *Stappia stellulata* 99.1% |
| 38 | YLY27 | *Idiomarina aestuarii* 99.4% |
| 39 | YLY28 | *Roseovarius halotolerans* 98.9% |
| 40 | YLY29 | *Shimia biformata* 97.8% |
| 41 | YLY32 | *Halomonas aestuarii* 96.8% |

Table S2. Hemolysis of strains

| Strain number | Hemolysis | Strain number | Hemolysis |
|---|---|---|---|
| M0101 | + | YLY06 | - |
| M0102 | - | YLY07 | - |
| M0103 | + | YLY08 | - |
| M0104 | + | YLY09 | - |
| M0106 | + | YLY10 | - |
| M0107 | + | YLY13 | + |
| ML1201 | + | YLY14 | + |
| ML1202 | + | YLY17 | + |
| ML1206 | - | YLY18 | + |
| ML1209 | + | YLY20 | - |
| ML1210 | - | YLY21 | - |
| ML1211 | + | YLY22 | + |
| ML1212 | + | YLY23 | + |
| ML1216 | + | YLY25 | - |
| ML1224 | + | YLY26 | - |
| ML1226 | + | YLY27 | - |
| ML1227 | + | YLY28 | - |
| ML1229 | - | YLY29 | + |
| YLY02 | - | YLY32 | - |
| YLY03 | - | SDUM002245 | + |
| YLY04 | - | LGG | - |
| YLY05 | - | | |

Note: "+" means positive; "-"stands means negative

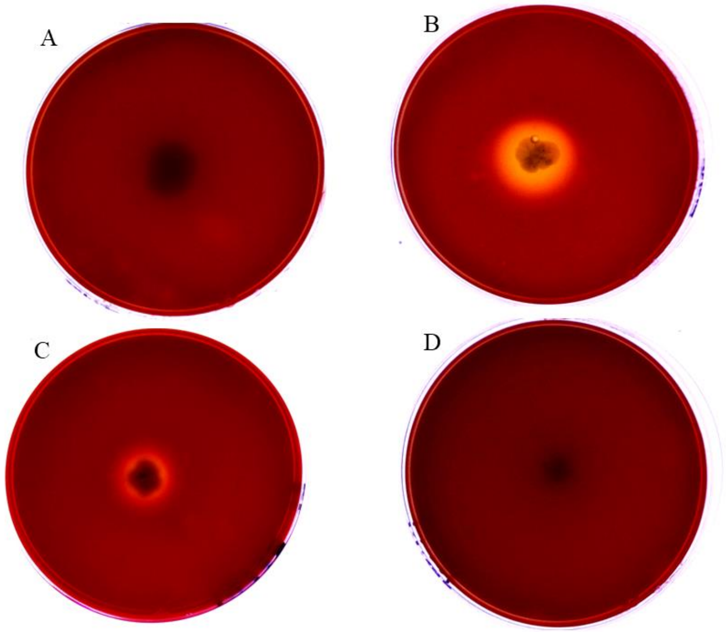


Figure S1. Hemolysis of some strains on 5% sheep blood agar (partial)

Note: A, strain ML1206; B, strain YLY14; C, control (*V. anguillarum* ); D, negative control (strain LGG)

Table S3 Low pH tolerance ability of twenty intestinal bacteria (Mean±SD)

| Strain number | $OD_{600}$ value | | Acid-resistant survival rate（%） |
|---|---|---|---|
| | pH 7.4 (pH5.6 for LGG) | pH3.0 | |

| | | | |
|---|---|---|---|
| M0102 | 0.49±0.03$^{cd,A}$ | 0.05±0.05$^{gh,B}$ | 10.30±0.02$^{gf}$ |
| ML1206 | 0.47±0.03$^{cde,A}$ | 0.37±0.02$^{c,A}$ | 79.51±0.03$^{b}$ |
| ML1210 | 0.41±0.02$^{defg,A}$ | 0.07±0.05$^{gh,A}$ | 16.33±0.02$^{gf}$ |
| ML1229 | 0.68±0.02$^{b,A}$ | 0.08±0.01$^{gh,A}$ | 11.80±0.02$^{gf}$ |
| YLY02 | 0.44±0.04$^{deg,A}$ | 0.06±0.00$^{gh,A}$ | 13.73±0.01$^{gf}$ |
| YLY03 | 0.81±0.02$^{a,A}$ | 0.09±0.02$^{g,A}$ | 11.12±0.02$^{gf}$ |
| YLY04 | 0.38±0.03$^{fg.A}$ | 0.07±0.01$^{gh,A}$ | 18.45±0.04$^{gf}$ |
| YLY05 | 0.45±0.02$^{de,A}$ | 0.28±0.03$^{e,A}$ | 61.14±0.05$^{e}$ |
| YLY06 | 0.68±0.01$^{b,A}$ | 0.13±0.01$^{f,A}$ | 18.69±0.02$^{gf}$ |
| YLY07 | 0.39±0.06$^{efg,A}$ | 0.06±0.01$^{gh,B}$ | 16.67±0.03$^{gf}$ |
| YLY08 | 0.20±0.01$^{j,A}$ | 0.05±0.01$^{i,A}$ | 23.89±0.04$^{f}$ |
| YLY09 | 0.38±0.03$^{gh,A}$ | 0.21±0.03$^{e,A}$ | 54.62±0.04$^{d}$ |
| YLY10 | 0.32±0.02$^{hi}$ | 0.17±0.01$^{e}$ | 53.77±0.05$^{d}$ |
| YLY20 | 0.43±0.02$^{defg}$ | 0.07±0.01$^{gh}$ | 16.91±0.01$^{gf}$ |
| YLY21 | 0.45±0.01$^{def}$ | 0.05±0.01$^{gh}$ | 11.77±0.01$^{gf}$ |
| YLY25 | 0.31±0.03$^{i,A}$ | 0.26±0.02$^{d,A}$ | 85.24±0.04$^{b}$ |
| YLY26 | 0.29±0.01$^{i,A}$ | 0.07±0.01$^{gh,A}$ | 25.02±0.02$^{f}$ |
| YLY27 | 0.30±0.02$^{i,A}$ | 0.07±0.00$^{gh,B}$ | 23.74±0.02$^{f}$ |
| YLY28 | 0.44±0.02$^{de,A}$ | 0.06±0.00$^{gh,B}$ | 13.57±0.01$^{gf}$ |
| YLY32 | 0.76±0.06$^{a,A}$ | 0.49±0.04$^{b,A}$ | 65.56±0.10$^{c}$ |
| LGG | 0.52±0.03$^{c,A}$ | 0.62±0.02$^{a,A}$ | 118.71±0.02$^{a}$ |

Note: different lowercase letters on superscript represent significant differences between different strains ($p< 0.05$); Different capital letters represent significant differences under different pH ($p<0.05$).

Table S4. Bile salt tolerance ability of twenty intestinal bacteria (Mean±SD)

| Strain number | $D_{600}$ value | | Bile salt tolerance survival rate（%） |
|---|---|---|---|
| | Bile salt 0.0% | Bile salt 0.3% | |
| M0102 | 0.41±0.08$^{ghij,A}$ | 0.07±0.01$^{ghi,B}$ | 17.75±0.03$^{efg}$ |
| ML1206 | 0.70±0.02$^{c,A}$ | 0.57±0.03$^{a,A}$ | 81.84±0.04$^{ab}$ |
| ML1210 | 0.46±0.04$^{fgh,A}$ | 0.07±0.01$^{ghi,B}$ | 15.96±0.01$^{efgh}$ |
| ML1229 | 0.46±0.02$^{fgh,A}$ | 0.06±0.01$^{hi,B}$ | 12.46±0.01$^{fgh}$ |
| YLY02 | 0.34±0.02$^{jkl,A}$ | 0.20±0.02$^{e,A}$ | 57.06±0.06$^{c}$ |
| YLY03 | 0.42±0.02$^{ghi,A}$ | 0.10±0.01$^{g,A}$ | 24.91±0.03$^{e}$ |
| YLY04 | 0.57±0.02$^{de,B}$ | 0.05±0.01$^{hi,B}$ | 9.29±0.01$^{h}$ |
| YLY05 | 0.51±0.01$^{ef,A}$ | 0.38±0.03$^{b,A}$ | 74.45±0.04$^{b}$ |
| YLY06 | 0.70±0.02$^{c,A}$ | 0.56±0.03$^{a,A}$ | 79.60±0.03$^{ab}$ |
| YLY07 | 0.32±0.02$^{l,B}$ | 0.04±0.01$^{hi,B}$ | 13.46±0.02$^{fgh}$ |
| YLY08 | 0.32±0.02$^{l,B}$ | 0.04±0.00$^{hi,B}$ | 12.56±0.01$^{fgh}$ |
| YLY09 | 0.43±0.02$^{gh,A}$ | 0.27±0.03$^{c,A}$ | 63.90±0.07$^{c}$ |
| YLY10 | 0.33±0.04$^{kl,A}$ | 0.04±0.01$^{i,B}$ | 11.32±0.03$^{fgh}$ |
| YLY20 | 0.39±0.03$^{hijkl,A}$ | 0.05±0.01$^{hi,B}$ | 13.01±0.03$^{fgh}$ |
| YLY21 | 0.40±0.02$^{ghijk,A}$ | 0.08±0.01$^{gh,B}$ | 20.16±0.03$^{ef}$ |
| YLY25 | 0.45±0.05$^{fgh,A}$ | 0.15±0.02$^{f,A}$ | 35.12±0.07$^{d}$ |
| YLY26 | 0.80±0.01$^{b,A}$ | 0.06±0.00$^{hi,B}$ | 7.50±0.01$^{h}$ |
| YLY27 | 0.60±0.01$^{d,A}$ | 0.04±0.00$^{hi,B}$ | 6.67±0.00$^{h}$ |
| YLY28 | 0.47±0.03$^{fg,A}$ | 0.05±0.01$^{hi,B}$ | 11.45±0.01$^{fgh}$ |
| YLY32 | 0.97±0.03$^{a,A}$ | 0.23±0.04$^{d,A}$ | 24.10±0.04$^{e}$ |
| LGG | 0.35±0.02$^{ijkl,A}$ | 0.30±0.02$^{c,A}$ | 87.42±0.05$^{a}$ |

Note: different lowercase letters on superscript represent significant differences between different strains ($p < 0.05$);

Different capital letters represent significant differences under different bile salt concentration values ($p<0.05$).

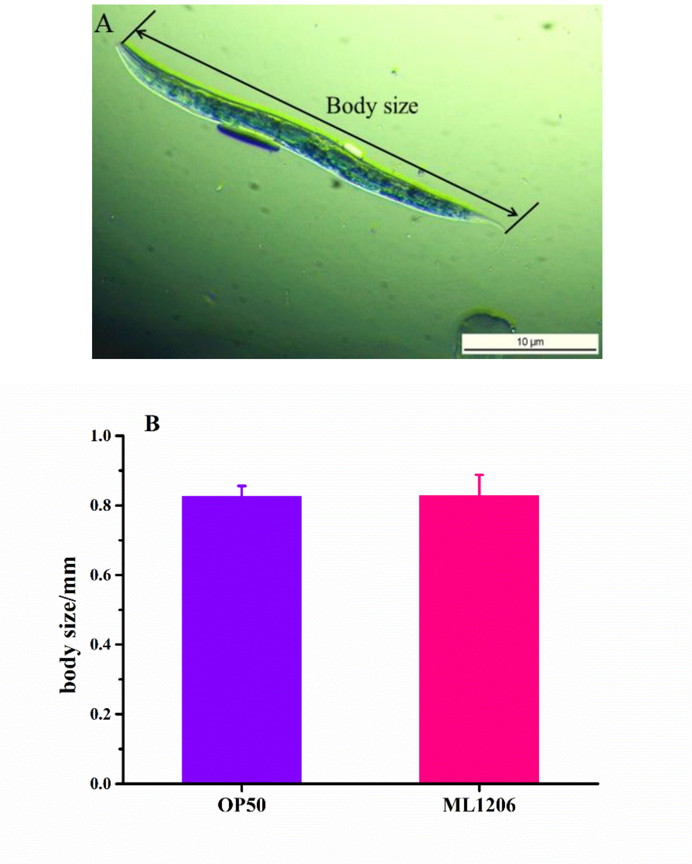


Figure S2. Effect of ML1206 strain on *C. elegans* growth: (a) Schematic diagram of *C. elegans* body length calculated with NIS elements software (b) Worms were grown in the presence of OP50 or ML1206 to the 2-day adult stage *C. elegans*. After anesthesia with levamisole hydrochloride, their body length was measured under a stereoscopic microscope (×400) with a micrometer. Data are representative of three independent experiments and presented as means ± SD.

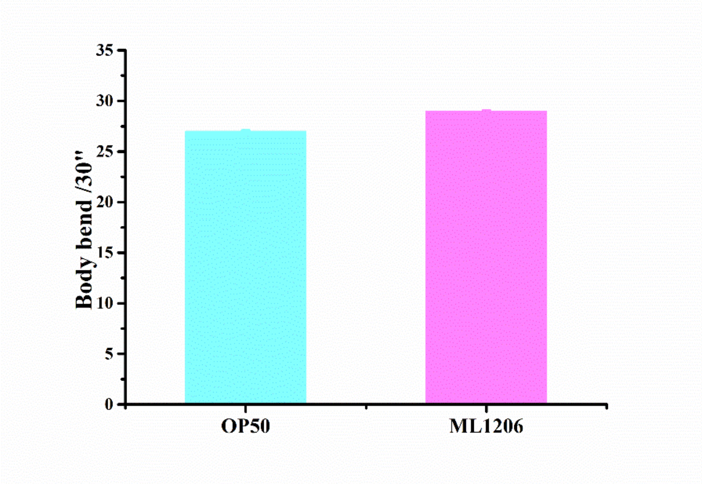


Figure S3. Effect of ML1206 strain on *C. elegans* body bending frequency: Worms were grown in the presence of OP50 or ML1206 to the 2-day adult stage *C. elegans*, and the body bending times of *C. elegans* within 30 S were recorded under a stereoscopic microscope (×400). Data are representative of three independent experiments and presented as means ± SD.

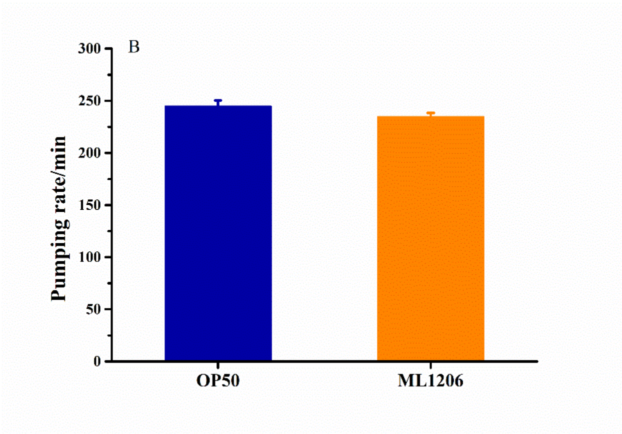


Figure S4. Effect of ML1206 strain on *C. elegans* pharyngeal pumping rate: Worms were grown in the presence of OP50 or ML1206 to the 2-day adult stage *C. elegans*. The worm was recorded within 1 min. Data are representative of three independent experiments and presented as means ± SD.

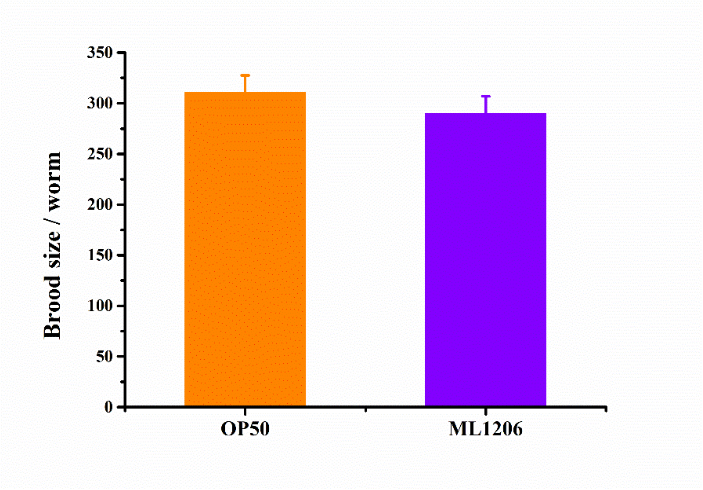


Figure S5. Effect of ML1206 strain on *C. elegans* reproduction ability: Average egg production per worm fed with OP50 or ML1206. Data are representative of three independent experiments and presented as means ± SD.


[1] Zanni E,; Laudenzi C,; Schifano E,; Palleschi C,; Perozzi G,; Uccelletti D,; Devirgiliis C. Impact of a Complex Food Microbiota on Energy Metabolism in the Model Organism *Caenorhabditis elegans*. *Biomed Res Int*. **2015**, 2015, 621709.

[2] Tsalik E.L,; Hobert O. Functional mapping of neurons that control locomotory behavior in *Caenorhabditis elegans*. *Journal of neurobiology*. **2003**, 56, 178-197.

[3] Wu C,; Wang H,; Zhang X.W,; Qin X.W,; Zhou X.H,; et al. L-arabinose promotes the growth and development of *Caenorhabditis elegans* and reduces fat synthesis. *Mod Food Sci Technol*. **2018**, 34, 1–6.
